# Empagliflozin Reverses Oxidized LDL-Induced RECK Suppression, Cardiotrophin-1 Expression, MMP Activation, and Human Aortic Smooth Muscle Cell Proliferation and Migration

**DOI:** 10.1155/2023/6112301

**Published:** 2023-10-04

**Authors:** Bysani Chandrasekar, Srinivas Mummidi, Vincent G. DeMarco, Yusuke Higashi

**Affiliations:** ^1^Research Service, Harry S. Truman Memorial Veterans Hospital, Columbia, MO, USA; ^2^Medicine, University of Missouri School of Medicine, Columbia, MO, USA; ^3^Medical Pharmacology and Physiology, University of Missouri, Columbia, MO, USA; ^4^Dalton Cardiovascular Center, University of Missouri, Columbia, MO, USA; ^5^Life Sciences, Texas A&M University-San Antonio, San Antonio, TX, USA; ^6^Medicine/Cardiology, Tulane University School of Medicine, New Orleans, LA, USA

## Abstract

Persistent oxidative stress and inflammation contribute causally to smooth muscle cell (SMC) proliferation and migration, the characteristic features of vascular proliferative diseases. Oxidatively modified low-density lipoproteins (OxLDL) elevate oxidative stress levels, inflammatory responses, and matrix metallopeptidase (MMP) activation, resulting ultimately in SMC migration, proliferation, and phenotype change. Reversion-inducing cysteine-rich protein with Kazal motifs (RECK) is a membrane-anchored MMP inhibitor. Empagliflozin is an SGLT2 inhibitor and exerts pleiotropic cardiovascular protective effects, including antioxidant and anti-inflammatory effects. Here, we investigated (i) whether OxLDL regulates RECK expression, (ii) whether ectopic expression of RECK reverses OxLDL-induced SMC migration and proliferation, and (iii) whether pretreatment with empagliflozin reverses OxLDL-induced RECK suppression, MMP activation, and SMC migration, proliferation, and differentiation. Indeed, results show that OxLDL at pathophysiological concentration promotes SMC migration and proliferation via NF-*κ*B/miR-30b-dependent RECK suppression. Moreover, OxLDL changed the SMC phenotype to a more pro-inflammatory type, and this effect is blunted by RECK overexpression. Further, treatment with empagliflozin reversed OxLDL-induced miR-30b induction, RECK suppression, MMP activation, SMC migration, proliferation, and proinflammatory phenotype changes. OxLDL-induced cardiotrophin (CT)-1 expression and CT-1 stimulated SMC proliferation and migration in part via leukemia inhibitory factor receptor (LIFR) and glycoprotein 130 (gp130). Ectopic expression of RECK inhibited these effects by physically associating with LIFR and gp130, as evidenced by immunoprecipitation/immunoblotting and double immunofluorescence. Importantly, empagliflozin inhibited CT-1-induced mitogenic and migratory effects. Together, these results suggest the therapeutic potential of sustaining RECK expression or empagliflozin in vascular diseases characterized by SMC proliferation and migration.

## 1. Introduction

Vascular proliferative diseases, such as restenosis following angioplasty, pulmonary arterial hypertension, and hypertensive vascular disease, are characterized by smooth muscle cell (SMC) migration, proliferation, and phenotype change. Persistent inflammation and oxidative stress both exert mitogenic and migratory effects in SMC. Oxidatively modified low-density lipoproteins (OxLDL) elevate oxidative stress levels and inflammatory responses in vascular SMCs [1], leading to their proliferation and migration, and the development and progression of vascular proliferative diseases [2, 3]. Thus, increased OxLDL is considered pathogenic in vascular proliferative diseases. Of note, various comorbid conditions, like hypertension, diabetes, hyperlipidemia, alcoholism, and smoking, exacerbate oxidative stress and serve as additional risk factors for vascular proliferative diseases.

Matrix metallopeptidases (MMPs), a family of zinc-dependent proteolytic enzymes that play a critical role in degrading the components of extracellular matrix, contribute to tissue homeostasis under physiological conditions and tissue degeneration and inflammation in pathologic conditions. The vascular SMCs express several MMPs [4], including the gelatinases MMP2 and MMP9. Both MMPs 2 and 9 have been shown to be upregulated in SMCs under conditions of elevated oxidative stress [5, 6]. In fact, both MMPs are suggested to serve as potential biomarkers for cardiovascular risk or mortality [7–9]. However, inhibition of MMP expression or activation by pharmacological inhibitors as a potential strategy to inhibit the development and progression of vascular proliferative diseases has met with clinical challenges, necessitating the development of newer strategies to target MMPs.

The expression of MMPs is regulated at multiple levels, including transcription, post-transcription, and enzymatic activity. Reversion-inducing cysteine-rich protein with Kazal motifs (RECK) is a glycosylphosphatidylinositol-anchored MMP inhibitor expressed in various cell types, including vascular SMCs [10–12]. We have previously reported that ectopic expression of RECK inhibits angiotensin-II (AngII)-induced MMP9 activation in cultured cardiac fibroblasts [13]. Ectopic expression of RECK also inhibited activation of AngII-induced MMP2 and MMP14 in those cells [13], suggesting that induction of RECK could serve as a negative regulator of pathologic remodeling and degeneration in vascular proliferative diseases mediated by elevated angiotensin II levels and hypertension. As such, we postulate that downregulation of RECK could contribute to oxidative stress-induced SMC proliferation and migration, and potentially vascular proliferative diseases.

Empagliflozin is an FDA-approved antihyperglycemic agent. It is a potent and highly selective inhibitor of sodium–glucose cotransporter-2 (SGLT-2) in renal proximal tubule epithelial cells, thereby blocking glucose reabsorption and promoting glucose excretion via urine. Intriguingly, several clinical trials have reported the pleiotropic beneficial effects of SGLT-2 inhibition on vascular function [14, 15], with the underlying mechanism postulated to be due, in part, to indirect effects mediated by improved diabetes status, such as weight loss, improved glycemic control, diuresis-induced blood volume reduction, as well as potential direct effects on the vasculature, such as improvements in endothelial function and vascular stiffness [16–18]. Empagliflozin has also been shown to blunt oxidative stress and inflammation [19]. We and others have shown that aortic SMCs express SGLT2 at a lower level than in the kidney [20, 21], suggesting that empagliflozin may have direct effects on vascular SMCs and exert nonglycemic vascular protective effects. Here, we hypothesized that empagliflozin has direct anti-inflammatory, antimitogenic, and antimigratory effects in SMCs exposed to OxLDL, a major pathogenic factor in vascular proliferative diseases. Specifically, we hypothesize that OxLDL downregulates RECK, an MMP regulator, in SMCs via oxidative stress-dependent and pro-inflammatory signaling pathways, leading to SMC migration, proliferation, and phenotype change, and empagliflozin counters these effects. Suggesting the therapeutic potential of sustaining RECK expression or empagliflozin in vascular diseases characterized by SMC migration, proliferation, and phenotype change.

## 2. Materials and Methods

### 2.1. Reagents

Human medium OxLDL (#770202) and native LDL (nLDL) (#770200) were purchased from KALEN Biomedical, LLC (Montgomery Village, MD) and have been previously described [22, 23]. Biologically active recombinant human cardiotrophin-1 (CT-1; #300-32) was purchased from PeproTech (Cranbury, NJ). Empagliflozin (EMPA; 1-chloro-4-(*β*-D-glucopyranos-1-yl)-2-[4-((S)-tetrahydrofuran-3-yl-oxy)-benzyl]-benzene; #S8022) was purchased from Selleckchem (Houston, TX), and used at 1 *μ*M for 15 min based on our previous published experience [14, 20, 24]. AR-100, a biphenylsulfonamide and selective inhibitor of MMP2, and AG-L-66085, a cell-permeable and reversible MMP9 inhibitor, were purchased from Santa Cruz Biotechnology, Inc. (Dallas, TX). Dimethylsulfoxide (DMSO) was purchased from EMD Biosciences (San Diego, CA). BioCoat™ Matrigel™ invasion chambers (#354481) were from BD/Discovery Labware (Bedford, MA). ITS-G (Insulin-Transferrin-Selenium, #41400045), SuperSignal® West Femto Maximum Sensitivity Substrate (#34096), Pierce™ BCA Protein Assay Kit (#23227), and protein molecular weight markers were all purchased from ThermoFisherScientific (Waltham, MA).

### 2.2. Cell Culture

Primary human aortic SMC, purchased from LONZA (CC-2571), were grown in SmGM-2 basal medium supplemented with SmGM™ −2 SingleQuots™ (LONZA, CC-4149) as reported previously [25]. Quantitative real-time PCR (RT-qPCR) using validated TaqMan® probes revealed that the cells were positive for *α* smooth muscle actin and smooth muscle myosin heavy chain and negative for Von Willebrand Factor (data not shown). When cells reached 70%–80% confluency, they were made quiescent by incubating in basal medium containing either 0.5% bovine serum albumin (BSA) or ITS-G 1X supplement for 48 hr and then exposed to OxLDL or nLDL at 45 *µ*g/ml for the indicated periods.

### 2.3. Adenoviral Vectors and Lentiviral Particles

The following adeno and lentiviral vectors were used: Ad.RECK (adenoviral vector expressing human RECK cDNA under the regulation of CMV promoter), Ad.GFP, Ad.siMMP2, Ad.siMMP9, and Ad.siGFP were all described previously [26]. SMC was traduced with adenoviral vectors at a multiplicity of infection (moi) of 10 for 1 hr in basal medium and then switched to complete medium for 24 hr. Lentiviral shRNA targeting p65 subunit of NF-*κ*B (#sc-29410-V) and green fluorescent protein (GFP) (sc-45924-V) were purchased from Santa Cruz Biotechnology, Inc. and have been described previously [23]. Lentivirus expression validated human glycoprotein 130 (gp130) (interleukin 6 cytokine family signal transducer/IL6ST; TRCN0000058284, target sequence: CCAGTCCAGATATTTCACATT) and leukemia inhibitory factor receptor (LIFR) (TRCN0000058769, target sequence: CCACCCATCATTGAGGAAGAA) were purchased from Sigma-Aldrich. SMCs at 50%–60% confluency were infected with the indicated lentiviral vector at a moi of 0.5 for 48 hr in complete medium. To enhance lentiviral transduction, SMC was cotreated with the cationic polymer Polybrene® (5 *μ*g/ml in water). Neither viral transduction nor the cationic polybrene Polybrene® modulated SMC shape, viability, or adherence (determined by trypan blue-dye exclusion; data not shown).

### 2.4. mRNA Expression

mRNA expression was analyzed by RT-qPCR. Total RNA free from DNA was isolated using Qiagen RNeasy Plus Micro Kit (#74034; Germantown, MD). The quality of RNA was analyzed by Agilent 2100 Bioanalyzer (Agilent Technologies, Palo Alto, CA). The RNA samples with an RNA integrity greater than 9.0 (scale = 1–10) were converted to cDNA and used for mRNA expression by RT-qPCR and TaqMan™ probes from Applied Biosystems™: RECK (Assay ID: Hs01019185_m1), 18s rRNA (Hs03003631-g1), miR-30b-5p (miR-30b, Assay ID: 000602), U6 (Assay ID: 001973), and CT-1 (Ctf1; Hs01106546_m1). No template controls were used in each assay, and samples processed without the RT served as a negative control. The cDNA samples were run in triplicate and analyzed using the 2^−*ΔΔ*Ct^ method. The mRNA expression data were normalized to corresponding 18s rRNA or U6 snRNA expression. The data were presented as fold change from untreated control.

### 2.5. miRNA Expression, Mimics, Inhibitors, Controls, and Transfections

To quantify miR-30b-5p expression, total RNA enriched with small RNA was extracted using the *mir*Vana™ miRNA Isolation Kit (Thermo Fisher Scientific) and analyzed by RT-qPCR using miRNA TaqMan® probe (Assay ID: 000602, miRbase accession# MI0000441, mature miRNA sequence: UGUAAACAUCCUACACUCAGCU) from Applied Biosystems/Thermo Fisher Scientific). mir-30b-5p mimic (Assay ID: MC10986), miRNA mimic negative control (#4464058), miR-30b-5p inhibitor (Assay ID: MH10986), Mir-30b-5p inhibitor negative control (#4464076) were all purchased from Applied Biosystems/Thermo Fisher Scientific. SMCs were transfected with 80 nM of the indicated mimic, mimic negative control, miRNA inhibitor, or inhibitor negative control using the Neon® transfection system (MPK-5000; Invitrogen, Waltham, MA). SMCs were microporated using the following parameters: pulse voltage: 1,300 V; pulse width: 20 ms; pulse number: 2; the tip type: 10 *μ*l) and then cultured for 24 hr. The transfection efficiency was ∼51% with 2% cell death as determined using the pEGFP-N1 vector (#6081-5; addgene). Transfections with the indicated mimics and inhibitors did not significantly modulate SMC shape, viability, or adherence (trypan blue dye exclusion; data not shown).

### 2.6. Protein Expression, Secretion, and Activity

Protein extraction, western blotting using 20 *μ*g of cleared whole cell homogenates, and detection of immunoreactive bands by enhanced chemiluminescence (ECL Plus; GE Healthcare) were all previously described [20, 22, 23, 26]. Densitometry to quantify the intensity of immunoreactive bands was also previously described. The following primary antibodies were used: RECK (1 : 1,000; catalog# 3433, Cell Signaling Technology, Inc./CST), *α*-Tubulin (1 : 1,000; #2144, CST), NF-*κ*B p65 (1 : 1,000; #3033; CST), MMP2 (1 : 500; ab97779, Abcam), MMP9 (1 : 1,000; #2270, CST), polyclonal human LIFR (used in neutralization (10 *µ*g/ml) and western blotting (0.1 *µ*g/ml)), #AF-249-NA, R&D Systems, Minneapolis, MN), and polyclonal antihuman gp130 (used in neutralization (2.5 *µ*g/ml) and Western blotting (1 *µ*g/ml)), #AF-228-NA, R&D Systems, Minneapolis, MN), control IgG (normal goat IgG control, #AF-108-C, R&D Systems). Secreted CT-1 levels in equal amounts of culture supernatants were quantified by a sandwich ELISA kit (sensitivity: 3.2 pg/ml, range 23.44–1,500 pg/ml; #ab216166, Abcam) according to the manufacturer's instructions. MMP2 activity was analyzed by the Sensolyte® Plus 520 MMP2 Fluorimetric and Enhances Selectivity Assay Kit (#AS-72224, AnaSpec, Fremont, CA) according to the manufacturer's instructions. MMP9 activity was analyzed by the Sensolyte® Plus 520 MMP-9 Fluorimetric and Enhanced Selectivity Assay Kit (#AS-72017, AnaSpec).

### 2.7. Immunoprecipitation (IP), Immunoblotting (IB), and Double Immunofluorescence/Confocal Microscopy

The physical association between RECK and LIFR or gp130 was analyzed by two different but complementary methods: IP/IB as previously described [27, 28] and double immunofluorescence. For IP, equal amounts of whole cell extracts were incubated with anti-RECK antibodies immobilized on agarose beads under slow rotation. After washing in a buffer containing 50 mM Tris–Cl, 150 mM NaCl, and 0.1% Nonidet P-40 three times, the bound proteins were eluted by boiling in SDS sample buffer, run on SDS–PAGE and IB with LIFR or gp130 antibodies. The antibodies against RECK, LIFR, and gp130 are described above. For immunofluorescence, cells seeded at a density of 2,000 cells on a glass slide were fixed with 4% paraformaldehyde and permeabilized with 0.25% Triton-X100 (30 min at 4°C), washed and then incubated with anti-RECK and anti-LIFR or anti-gp130 antibodies (1 hr at RT) followed by secondary antibodies conjugated with Alexa flour 488 or Alexa flour 594 antibodies (Abcam) and nuclear localizing DAPI (4′,6-diamidino-2-phenylindole dihydrochloride, #D1306, ThermoFisherScientific) at RT for 1 hr. Omission of primary antibodies failed to provide specific signals and served as controls. Images were captured by confocal microscopy (Leica Microsystems, Wetzlar, Germany). Red and green signals were colocalized along with DAPI (blue) in merged images.

### 2.8. Cell Proliferation

SMC proliferation was analyzed according to the manufacturer's instruction using the CyQUANT GR dye assay (Molecular Probes, Eugene, OR) as previously described [25, 26, 29]. SMCs were seeded in 96-well clear bottom, black-sided flat-bottom plates (VWR Scientific Products, West Chester, PA) at 1 × 10^3^ cells/well containing 200 *μ*l of complete medium and incubated for 24 hr. The complete medium was then replaced with medium containing 0.5% BSA (conditioning medium) and incubated for an additional 48 hr (quiescence). The quiescent cells were then incubated with OxLDL or nLDL at indicated concentrations for 48 hr. The medium was then removed, and plates were frozen at −80°C for 2 hr prior to assay. The plates were then thawed, stained with CyQUANT GR dye according to the manufacturer's protocol and read on a FLX800 microplate fluorescence reader (Bio-Tek Instruments, Winooski, VT) at 485/20 excitation and 528/20 emission, and analyzed using the KC^4^ software (Bio-Tek Instruments).

### 2.9. Cell Migration

The effects of OxLDL on SMC migration were analyzed using BioCoat™ Matrigel™ invasion chambers and 8.0 *μ*m pore polyethylene terephthalate membranes with a thin layer of Matrigel™ basement membrane matrix and has been previously described [25, 26, 30, 31]. In brief, following trypsinization, SMCs were suspended in a medium containing 0.5% BSA and no fetal bovine serum, and 1 ml containing 2.0 × 10^5^ cells/ml was layered on the coated insert filters. The cells were then stimulated with OxLDL or nLDL at 45 *µ*g/ml. The lower chamber contained 20% serum. The plates were then incubated at 37°C for 18 hr. After washing the membranes in phosphate-buffered saline, noninvading cells on the upper surface were removed using cotton swabs and stained with hematoxylin. SMCs migrating to the lower surface of the membrane were counted at 20× magnification in 10 independent fields and summarized as mean ± SEM.

### 2.10. Phenotypic Modulation

To determine whether OxLDL induces SMC phenotype change and whether this effect is modulated by RECK overexpression, low passage SMCs were infected with Ad.RECK or Ad.GFP at moi 10 for 24 hr, and then incubated with 45 *μ*g/ml OxLDL or nLDL for 48 hr. Total RNA was isolated and analyzed for the expression levels of SMC markers ACTA2 (actin alpha 2, smooth muscle; assay ID: Hs00426825_g1 alpha smooth muscle actin) and MYH11 (myosin heavy chain 11; assay ID: Hs00975796_m1), and the proinflammatory markers ICAM1 (intercellular adhesion molecule 1; assay ID: Hs00164932_m1), Galectin 3 (LGALS3; assay ID: Hs03680062_m1), and Olr1 (oxidized low-density lipoprotein receptor 1; assay ID: Hs01552593_m1) by RT-qPCR using recommended TaqMan® probes from Applied Biosciences, and analyzed using the 2^−*ΔΔ*Ct^ method. The mRNA expressions data were normalized to corresponding *β*-actin (actin-beta; assay ID: Hs01060665_g1) expression, presented as fold change from untreated control.

### 2.11. Statistical Analysis

All data are expressed as means ± SEM. Statistical analysis was performed using GraphPad Prism 8 software (San Diego, CA). Statistical significance was determined by a two-way analysis of variance, followed by Fisher's least significant difference test and Bonferroni's post hoc analysis for multiple comparisons. For single comparisons, a unpaired two-tailed Student's *t*-test was employed. Prior to performing statistical analysis, normality of distribution as well as equality of variance were considered. For example, in all mRNA expression analyses, we had a similar number of replicates. No samples were deleted. Differences are considered significant if the *P* value is <0.05. The figures contained a representative image of western blot analysis from three to four independent experiments, and the densitometric data shown at the bottom or sides contained semiquantification of the intensity of immunoreactive bands as fold changes over control, which was set at a value of 1.

## 3. Results

### 3.1. OxLDL Suppresses RECK Expression in Primary Human Aortic SMC

**Figure 1:**
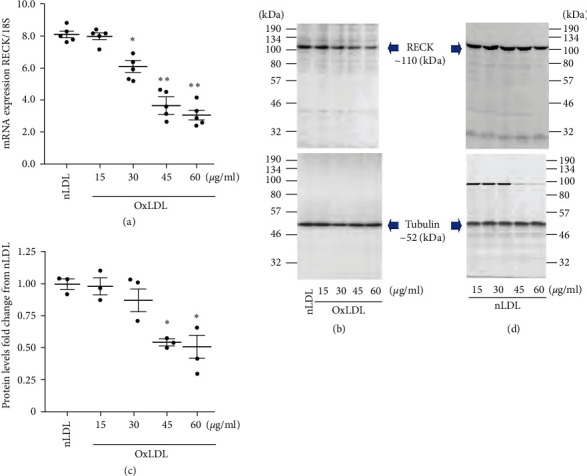
Oxidized low-density lipoprotein (OxLDL) suppresses RECK expression in primary human aortic smooth muscle cells (SMC). (a–c) OxLDL, but not nLDL, suppresses RECK mRNA expression in a dose-dependent manner. Quiescent SMCs were exposed to OxLDL at indicated concentrations for 2 hr. RECK expression was analyzed by RT-qPCR using a validated TaqMan™ probe (a; *n* = 5). Native LDL (nLDL, 45 *µ*g/ml) served as a control. 18s served as an invariant control. The mRNA expression was presented as a ratio of RECK mRNA to corresponding 18s rRNA. RECK protein levels were analyzed by Western blotting (b, *n* = 3). Tubulin served as an invariant control. Intensity of immunoreactive bands from three independent experiments was semiquantified by densitometry, and the ratio of RECK to corresponding Tubulin was presented as fold change from nLDL. (d) nLDL up to 60 mg/ml had no significant effects on basal RECK expression. Quiescent SMC incubated with nLDL up to 60 mg/ml for 2 hr were analyzed by western blotting, and a representative image of three independent experiments is shown. (a)  ^*∗*^*P* < 0.05,  ^*∗∗*^*P* < 0.01 versus nLDL (*n* = 5), (b)  ^*∗*^*P* < 0.05 versus nLDL (*n* = 3).

Atherosclerosis is a chronic inflammatory disease and is characterized by persistent low-grade oxidative stress. Increased accumulation of OxLDL in the subendothelial space of arteries plays a causative role in atherosclerosis by promoting SMC migration and proliferation. RECK is a membrane-anchored inhibitor of matrix-degrading metalloproteinases and inflammation, suggesting that its increased expression may have protective effects in atherosclerosis. However, the effect of OxLDL on RECK expression in SMCs is not known. Therefore, we incubated quiescent SMC with OxLDL at various concentrations ranging from 15 to 60 *μ*g/ml for 2 hr. nLDL served as a control. The results show that exposure to nLDL at various concentrations did not affect RECK expression (data not shown). However, OxLDL suppressed RECK expression in a dose-dependent manner, with similar levels of suppression seen at both 45 and 60 *µ*g/ml (Figure 1(a)). At these concentrations, OxLDL also suppressed RECK protein expression (Figure 1(b); densitometric results from three independent experiments are summarized in Figure 1(c)). Once again, the suppressive effects of OxLDL on RECK protein expression were similar at both 45 and 60 *µ*g/ml. Therefore, in all subsequent experiments, OxLDL was used at a concentration of 45 *µ*g/ml. Importantly, nLDL up to 60 *μ*g/ml had no significant effect on RECK protein levels (Figure 1(d)). These results indicate for the first time that OxLDL suppresses RECK expression in SMCs (Figure 1).

### 3.2. OxLDL Induces SMC Migration by Stimulating MMP2 and MMP9 Activation

**Figure 2:**
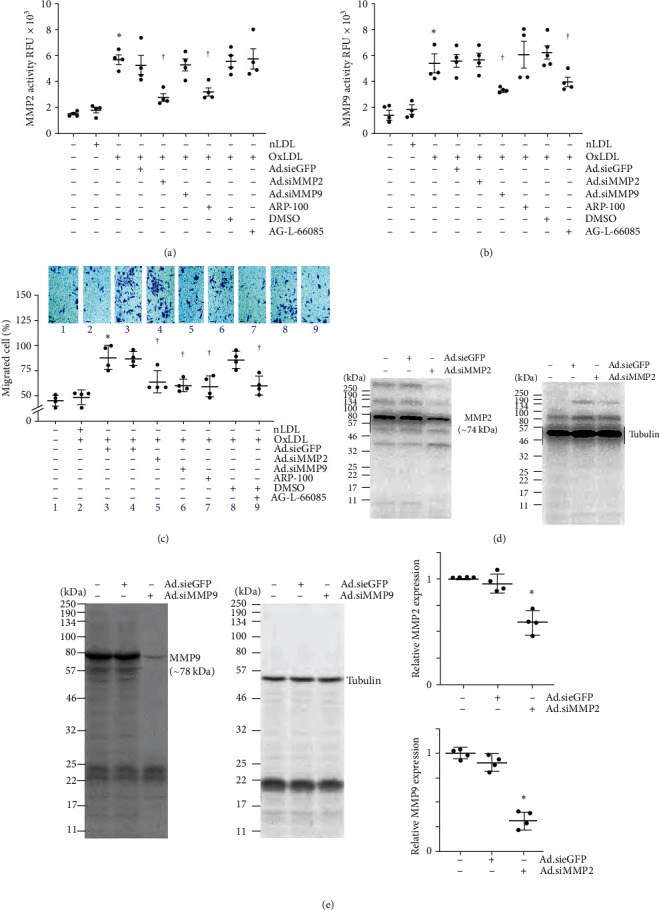
OxLDL stimulates SMC migration via MMP2 and MMP9 activation. (a, b) OxLDL stimulates MMP2 and MMP9 activation. SMC made quiescent in medium containing ITS-G 1x supplement were exposed to OxLDL or nLDL for 24 hr and then analyzed for MMP2 and MMP9 activation using SensoLyte® 520 fluorimetric assays. In a subset of experiments, SMCs transduced with adenovirus expressing siRNA against MMP2 (Ad.siMMP2), MMP9 (Ad.siMMP9), or eGFP (Ad.sieGFP) were made quiescent and then exposed to either OxLDL or nLDL. In another set of samples, quiescent SMCs were treated with inhibitors of MMP2 or MMP9 prior to OxLDL exposure. (c) Targeting MMP2 or MMP9 also inhibits OxLDL-induced SMC migration analyzed by the Boyden chamber assay. SMCs migrating to the lower surface of the membrane were counted at 20x magnification in 10 independent fields and summarized as mean ± SEM. The inset shows representative images of Matrigel™ transwell invasion. (d, e) Knockdown of MMP2 (d) and MMP9 (e) was confirmed by western blotting and densitometric results are presented on the right of panel E. (a, b)  ^*∗*^*P* < 0.01 versus untreated, ^†^*P*< at least 0.05 versus nLDL or untreated or DMSO (*n* = 4), (c)  ^*∗*^*P* < 0.01 versus untreated, ^†^*P* < 0.05 versus nLDL or untreated or DMSO (*n* = 4), (e)  ^*∗*^*P* < 0.05 versus eGFP siRNA (*n* = 3).

Activation of MMP2 and MMP9 has been shown to contribute to SMC migration in vitro and intimal hyperplasia in vivo. Both MMPs are known to degrade extracellular matrix. Therefore, SMC made quiescent in ITS-G (1x) supplement for 48 hr were incubated with OxLDL or nLDL at 45 mg/ml for 24 hr and then analyzed using a highly sensitive fluorometric assay. The results show that, at the indicated concentration, OxLDL induces significant activation of MMP2 (Figure 2(a)) in SMC. Further, knockdown of MMP2 using an adenoviral vector expressing MMP2 siRNA (Ad.siMMP2, moi 100 for 48 hr) and pretreatment with the MMP2-specific inhibitor ARP-100 (5 *μ*M for 12 hr), each significantly attenuated OxLDL-induced MMP2 activation (Figure 2(a)). Similarly, the knockdown of MMP9 by Ad.siMMP9 and incubation with MMP9-specific inhibitor AG-L-66085 (5 *μ*M for 12 hr) attenuated OxLDL-induced MMP9 activation (Figure 2(b)). Transduction with Ad.siGFP affected neither MMP2 nor MMP9 activity. Similarly, DMSO used as a solvent control, failed to affect MMP2 or MMP9 activity. Importantly, targeting MMP2 and MMP9 by adenoviral transduction of specific siRNA or pharmacological inhibitors each blunted OxLDL-induced SMC migration (Figure 2(c); the inset shows representative images of Matrigel™ transwell invasion). Knockdown of MMP2 (Figure 2(d)) and MMP9 (Figure 2(e)) was confirmed by western blotting, and densitometric results from four independent experiments are shown in the right-hand panels. Together, these results indicate that OxLDL stimulates SMC migration in part via MMP2 and MMP9 (Figure 2).

### 3.3. Ectopic Expression of RECK Inhibits OxLDL-Induced SMC Proliferation, Migration, and the Proinflammatory Phenotype

**Figure 3:**
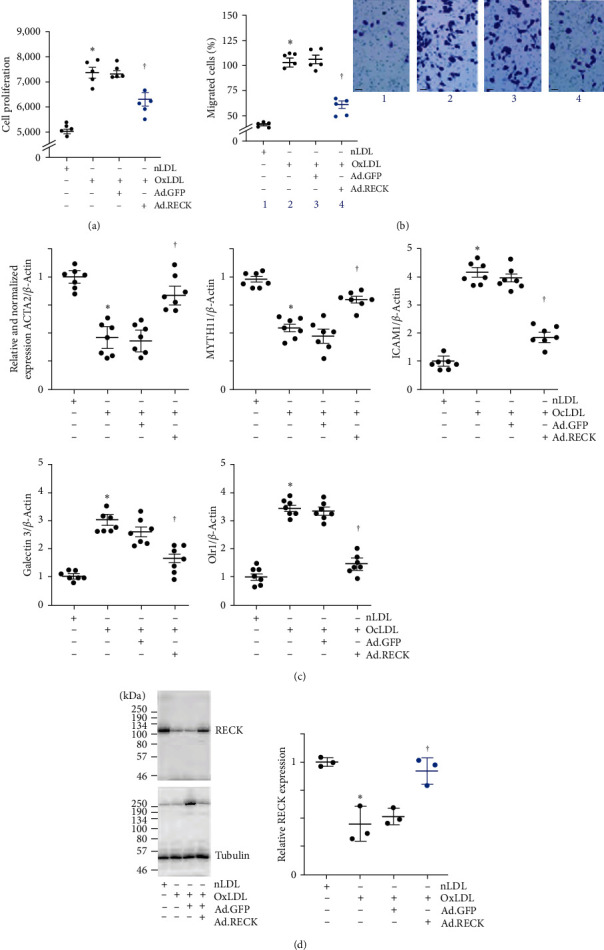
Ectopic expression of RECK blunts OxLDL-induced SMC proliferation and migration. (a, b) Pathophysiological concentrations of OxLDL induce SMC proliferation (a) as analyzed by the CyQUANT GR dye assay and migration (b) analyzed by the Boyden chamber assay. SMCs migrating to the lower surface of the membrane were counted at 20x magnification in 10 independent fields and summarized as mean ± SEM. The inset shows representative images of Matrigel™ transwell invasion. (c) OxLDL induced proinflammatory phenotype changes in SMC. SMC transduced with Ad.RECK or Ad.GFP (moi10 for 24 hr) were exposed to OxLDL (45 mg/ml for 48 hr) and analyzed for SMC markers *α*SMA and MYH11, and proinflammatory markers ICAM1, Galectin 3, and Olr1. (d) OxLDL does not inhibit ectopically expressed RECK. SMCs transduced with Ad.RECK were made quiescent, exposed to OxLDL for 48 hr, and analyzed for RECK expression by western blotting. (a)  ^*∗*^*P* < 0.01 versus nLDL, ^†^*P* < 0.05 versus Ad.RECK (*n* = 5), (b)  ^*∗*^*P* < 0.01 versus nLDL, ^†^*P* < 0.05 versus. Ad.RECK (*n* = 5), (c)  ^*∗*^*P* < 0.01 versus nLDL, ^†^*P* < 0.05 versus OxLDL ± Ad.GFP, (*n* = 5), (d)  ^*∗*^*P* < 0.05 versus nLDL, ^†^*P* < 0.05 versus OxLDL or OxLDL + Ad.GFP (*n* = 3).

The proliferation and migration of SMCs contribute to vessel wall thickening and luminal occlusion during restenosis [32]. Moreover, SMC shows phenotype modulation. Since OxLDL suppressed RECK expression (Figure 1), we hypothesized that its increased expression blunts the promitogenic and promigratory effects of OxLDL on SMCs. Moreover, we hypothesized that RECK overexpression blunts SMC phenotype change. The CyQUANT GR assay and BioCoat™ Matrigel™ invasion chamber assay revealed that exposure to OxLDL at 45 *μ*g/ml stimulated SMC proliferation (Figure 3(a)) and migration (Figure 3(b)), respectively, and these effects were blunted by the ectopic expression of RECK by adenoviral transduction (Ad.RECK at moi10 for 24 hr) (Figures 3(a) and 3(b), the inset in Figure 3(b) shows representative images of Matrigel™ transwell invasion). Moreover, OxLDL induced a proinflammatory phenotype change in SMC, as evidenced by the reduced expression of SMC markers *α*SMA and MYH11, and increased the expression of proinflammatory markers ICAM1, Galectin 3 and Olr1 (Figure 3(c)). Importantly, RECK overexpression reversed these effects. Ectopic expression of GFP, however, had no significant effect on proliferation, migration, and proinflammatory phenotype changes. Importantly, OxLDL had no suppressive effects on ectopically expressed RECK expression (Figure 3(d) shows a representative western blotting and densitometric results summarized from three independent experiments are shown in the right-hand panel). Together, these results indicate that ectopic expression of RECK blunts OxLDL-induced SMC proliferation, migration, and proinflammatory phenotype changes (Figure 3).

### 3.4. OxLDL/CT-1-Mediated SMC Proliferation and Migration Are Inhibited by Ectopic Expression of RECK

**Figure 4:**
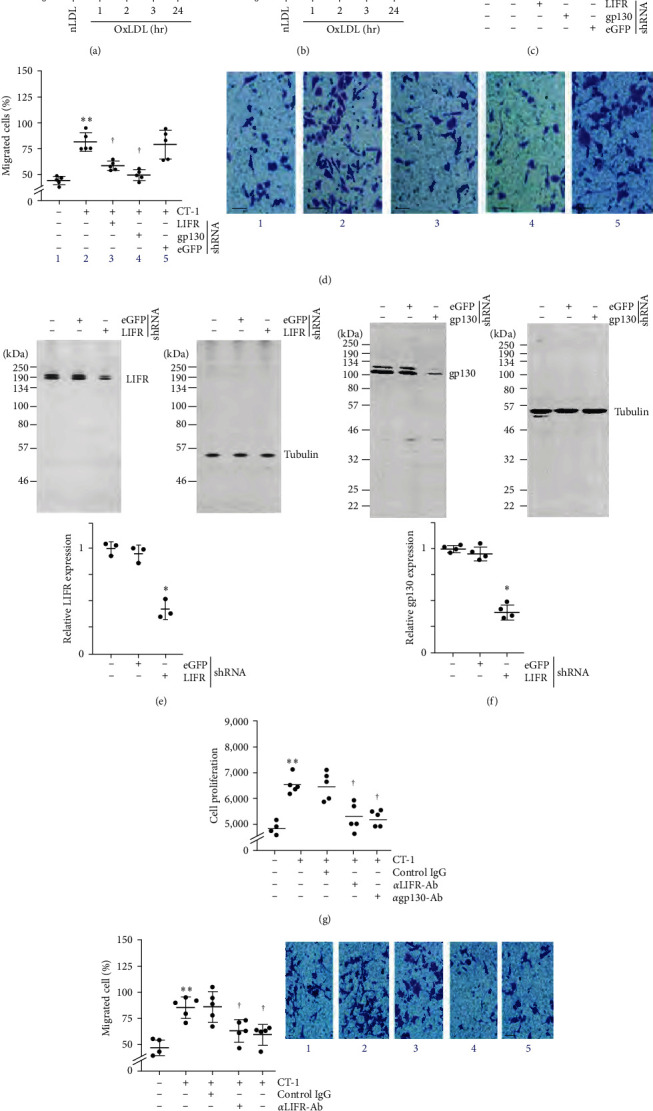
OxLDL stimulates SMC migration and proliferation via CT-1 induction. (a, b) OxLDL stimulates CT-1 mRNA expression and secretion. Quiescent SMCs treated with OxLDL for the indicated periods were analyzed for CT-1 mRNA expression by RT-qPCR and its secreted levels in equal amounts of culture supernatants by ELISA. nLDL served as a control. (c–f) CT-1 stimulates SMC migration and proliferation via LIFR and gp130. SMCs were transduced with validated lentiviral LIFR or gp130 shRNA, made quiescent, and exposed to CT-1. Cell proliferation after 48 hr (c) and migration after 18 hr (d) were analyzed by CyQUANT GR dye assay and Boyden chamber assay, respectively. The inset in (d) shows representative images of Matrigel™ transwell invasion. Knockdown of LIFR and gp130 was confirmed by western blotting (e, f), and summarized semiquantification of the intensity of immunoreactive bands is shown in the lower panels. (g, h), Preincubation with neutralizing anti-LIFR or anti-gp130 antibodies blunt CT-1-induced SMC proliferation and migration. The inset in (h) shows representative images of Matrigel™ transwell invasion. (a–d, g, h)  ^*∗*^0.05,  ^*∗∗*^*P* < 0.01 versus nLDL (*n* = 4 or 5); (e, f)  ^*∗*^*P* < 0.05 versus eGFP shRNA (*n* = 3).

OxLDL exerts proinflammatory effects by inducing the expression of various oxidative stress-responsive proinflammatory cytokines [33]. CTF1 or CT-1 is a member of the interleukin-6 family, and its increased expression has been shown to be associate with the development and progression of atherosclerosis. Here, we investigated whether OxLDL induces CT-1 expression in SMC. Results show that exposure to OxLDL at 45 *μ*g/ml upregulated CT-1 mRNA expression in a time-dependent manner, with a significant increase detected as early as 1 hr (Figure 4(a)). Its levels increased further at 2 and 3 hr and declined by 24 hr but remained high compared to basal levels (Figure 4(a)). Supporting these data, quantification of secreted CT-1 levels by ELISA using equal amounts of cultured supernatants showed its increased levels at 2 hr, which peaked at 24 hr (Figure 4(b)). Further, exposure to functionally active, recombinant human CT-1, at pathophysiological concentrations (1 ng/ml) [34], stimulated SMC proliferation (Figure 4(c)) and migration (Figure 4(d), the inset shows representative images of Matrigel™ transwell invasion) at 48 and 18 hr, respectively, and these effects were blunted by silencing LIFR and the signal transducer gp130 by lentiviral transduction of specific shRNA. Representative Western blots using 20 *μ*g of whole cell lysates demonstrated LIFR and gp130 knockdown (Figures 4(e) and 4(f), respectively). The densitometric data from three to four independent experiments are summarized at the bottom. Confirming the results obtained with LIFR and gp130 knockdown, preincubation with neutralizing LIFR and gp130 antibodies inhibited CT-1-induced SMC proliferation (Figure 4(g)) and migration (Figure 4(h), the inset shows representative images of Matrigel™ transwell invasion). Together, these results indicate that OxLDL induces CT-1 expression, and CT-1 stimulates SMC proliferation and migration in LIFR- and gp130-dependent manner (Figure 4).

### 3.5. RECK Physically Associates with LIFR and gp130

**Figure 5:**
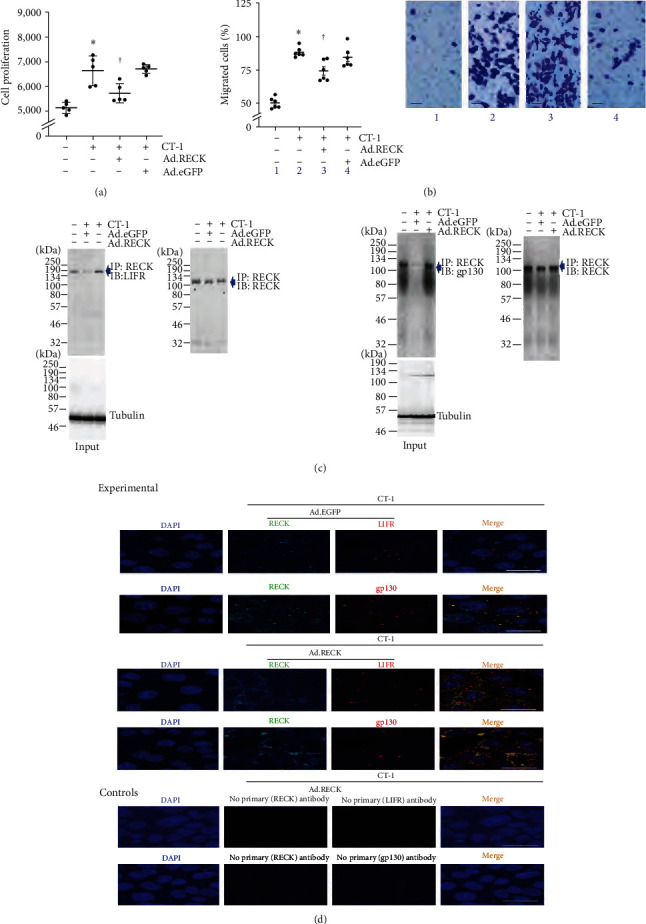
Ectopic expression of RECK blunts CT-1-induced SMC proliferation and migration potentially by associating physically with LIFR and gp130. (a, b) Ectopic expression of RECK blunts CT-1-induced SMC proliferation (a) and migration (b). Ad. GFP served as a control. SMC transduced with Ad.RECK or Ad.GFP was made quiescent, exposed to CT-1 for 48 hr (g) or 18 hr (h), and analyzed for proliferation or migration by CyQUANT GR dye assay and Boyden chamber assay, respectively. (c, d) CT-1 promotes RECK physical association with LIFT and gp130. SMCs transduced with Ad.RECK or Ad.GFP was exposed to CT-1 for 15 min and then immunoprecipitated (IP) with anti-RECK antibodies and immunoblotted (IB) with anti-LIFR (c) or gp130 (d) antibodies. Equal loading of immunoprecipitates was confirmed by blotting with anti-RECK antibodies (right-hand panels). (d) RECK (green) and LIFR or gp130 (red) physical association is analyzed by double immunofluorescence and confocal microscopy. The merged images (orange) show RECK, LIFR, or gp130 and nuclei. The omission of primary antibodies in the control panels served as a negative control. DAPI stains the nuclei blue. (a, b)  ^*∗*^*P* < 0.05 versus untreated, ^†^*P* < 0.05 CT-1 or CT-1 + Ad.GFP (*n* = 5–6).

Previously, RECK was shown to be physically associated with IL-6R and gp130 in breast cancer cells. However, whether this interaction had any biological significance in those cells was not investigated. We recently reported that ectopically expressed RECK-bound gp130 and blunted IL-6-induced SMC proliferation [35]. Since CT-1 signals via LIFR and gp130, we next investigated whether RECK physically associates with LIFR and gp130 and inhibits CT-1-induced SMC proliferation and migration. Results show that while CT-1 at 1 ng/ml stimulates SMC proliferation (Figure 5(a)) and migration (Figure 5(b), the inset shows representative images of Matrigel™ transwell invasion), and these effects were inhibited by ectopically expressed RECK by adenoviral transduction. Further, IP and IB revealed that RECK physically associates with LIFR (Figure 5(c), left) and gp130 (Figure 5(c), right) in SMC at basal conditions. While exposure to CT-1 reduced the binding, ectopic expression of RECK, but not eGFP, enhanced their binding. Similar levels of protein input have been shown in the bottom panels and demonstrate equal levels of Tubulin by IB (Panel C, bottom panels). Since IP/IB is semiquantitative, we also performed colocalization experiments by immunofluorescence using Alexa flour 488 (green) or Alexa flour 594 (red) tagged secondary antibodies with DAPI serving as a marker of nucleus (blue). The double immunofluorescence data confirmed the IP/IB experiments and demonstrated that ectopically expressed RECK binds LIFR and gp130 (Figure 5(d)). However, the omission of primary antibodies served as negative controls and showed no specific signals. Together, these IP/IB and double immunofluorescence experiments indicate that ectopically expressed RECK inhibits CT-1-induced SMC proliferation and migration, potentially by binding to LIFR and gp130 (Figure 5).

### 3.6. OxLDL Suppresses RECK Expression via miR-30b Induction

**Figure 6:**
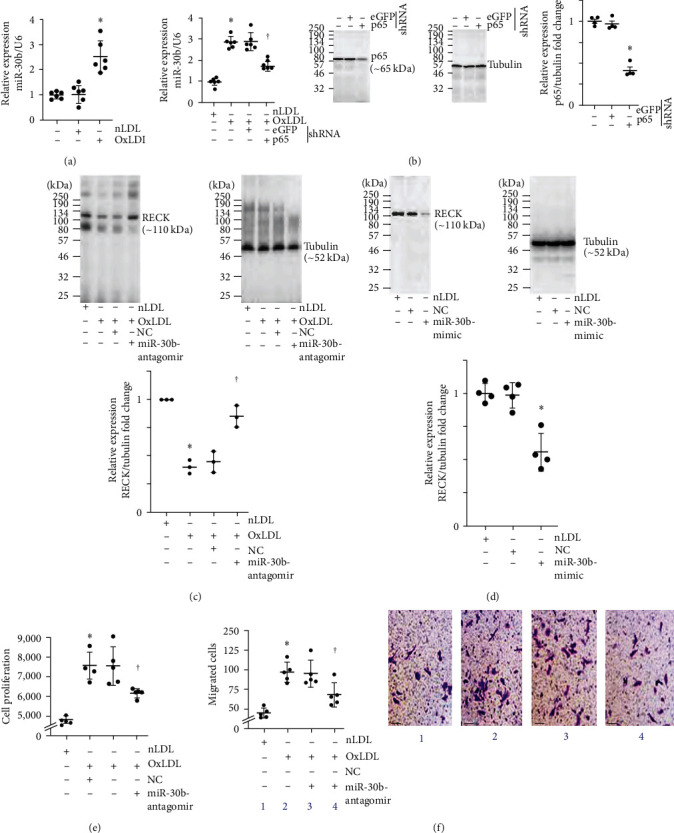
OxLDL suppresses RECK expression via NF-*κ*B-dependent miR-30b induction. (a) OxLDL upregulates miR-30b-5p (miR-30b) expression. Quiescent SMCs exposed to OxLDL or nLDL for 2 hr were analyzed for miR-30b expression by RT-qPCR using a TaqMan® probe. (b) OxLDL induces miR-30b expression in part via NF-*κ*B. SMCs transduced with lentiviral NF-*κ*B p65 or eGFP shRNA were made quiescent and then exposed to OxLDL or nLDL for 2 hr. miR-30b expression was analyzed by RT-qPCR. (c) miR-30b antagomir reverses OxLDL-induced RECK suppression. RECK expression was analyzed by western blotting, and the densitometric data is shown on the right. (d) miR-30b-mimic inhibits RECK expression. SMCs transfected with miR-30b-mimic or corresponding NC were analyzed for RECK expression by western blotting. Densitometric results are shown on the right. (e, f), miR-30b-antagomir attenuates OxLDL-induced SMC proliferation and migration. SMCs transfected with miR-30b-antagomir or corresponding NC were made quiescent and exposed to OxLDL or nLDL for 48 hr (e) or 18 hr (f) and analyzed for proliferation and migration. The inset in (f) shows representative images of Matrigel™ transwell invasion. (a, b)  ^*∗∗*^*P* < 0.01 versus nLDL, ^†^*P* < 0.05 versus OxLDL or OxLDL + eGFP (*n* = 6); (c, d)  ^*∗*^*P* < 0.05 versus nLDL, ^†^*P* < 0.05 versus OxLDL or OxLDL + NC (*n* = 3); (e, f)  ^*∗∗*^*P* < 0.01 versus nLDL, ^†^*P*< at least 0.05 versus OxLDL or OxLDL + NC (*n* = 5).

We have demonstrated that OxLDL suppresses RECK expression in SMC (Figure 1), and its ectopic overexpression blunts OxLDL-induced SMC proliferation and migration. However, the mechanisms underlying OxLDL-induced RECK suppression are not known. RECK expression is regulated at multiple levels, including at the post-transcriptional level. Multiple miRNAs have been shown experimentally to target RECK expression in various cell types. Since miR-30b is an oxidative stress-responsive miRNA [36], and OxLDL induces oxidative stress, we determined whether OxLDL induces miR-30b and investigated the mechanism underlying its induction and whether its miR-30b induction suppresses RECK expression in SMC and promotes cell proliferation and migration. Indeed, OxLDL, but not nLDL, at 45 *μ*g/ml induced miR-30b expression at a significant level (Figure 6(a)), and its induction was markedly inhibited by silencing p65 subunit of NF-*κ*B (Figure 6(b); knockdown of p65 was confirmed by western blotting and densitometric results from three independent experiments are summarized in the right-hand panel). Further, transfection with miR-30b antagomir at 80 nM concentration reversed OxLDL-induced RECK suppression (Figure 6(c), a representative Western blotting and densitometric results from three independent experiments are summarized in the right-hand panel). In contrast, transfection with miR-30b mimic by itself suppressed basal RECK expression (Figure 6(d); a representative western blot and densitometric results from three independent experiments are summarized in the right-hand panel), supporting the inhibitory effects of miR-30b on RECK expression. Importantly, transfection with miR-30b antagomir reversed OxLDL-induced SMC proliferation (Figure 6(e)) and migration (Figure 6(f), the inset shows representative images of Matrigel™ transwell invasion). Together, these results indicate that NF-*κ*B-dependent miR-30b mediates OxLDL-induced RECK suppression (Figure 6).

### 3.7. Empagliflozin Inhibits OxLDL-Induced miR-30b Expression, MMP Activation, and SMC Proliferation and Migration

**Figure 7:**
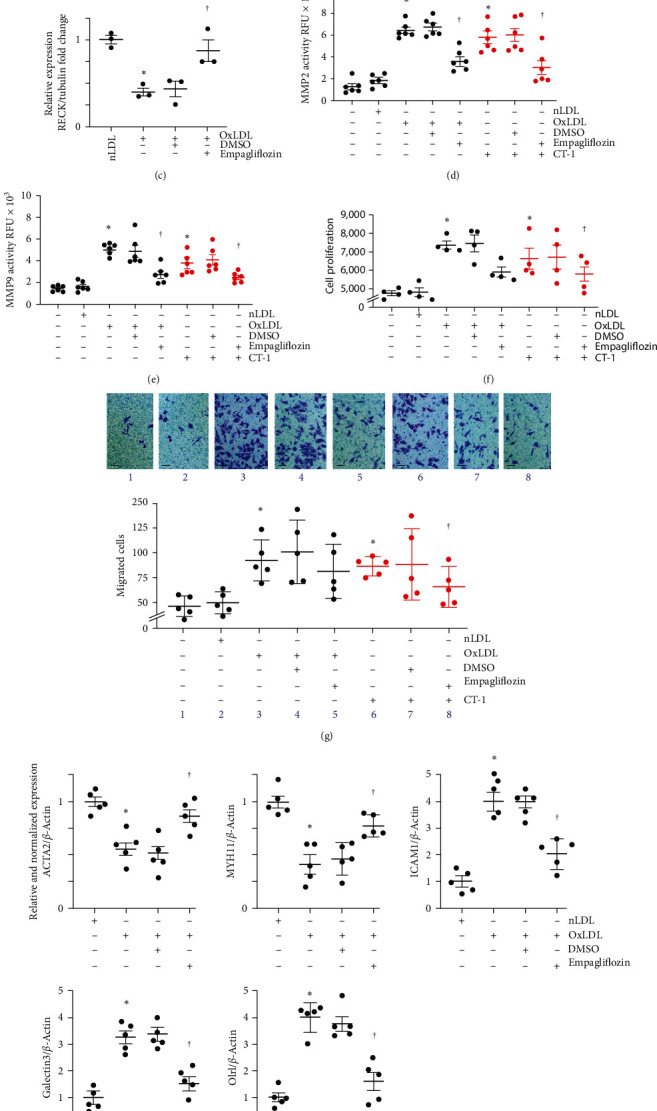
Empagliflozin inhibits OxLDL and CT-1-induced SMC proliferation and migration. (a) Empagliflozin inhibits OxLDL-induced miR-30b expression. Quiescent SMCs were treated with empagliflozin for 15 min followed by the addition of OxLDL or nLDL for 2 hr and analyzed for miR-30b expression by RT-qPCR using a TaqMan® probe. (b, c) Empagliflozin reverses OxLDL-induced RECK suppression. Quiescent SMCs were treated with empagliflozin as in (a), were exposed OxLDL or nLDL for 6 hr and analyzed for RECK expression by western blotting. Semiquantification of the intensity of immunoreactive bands by densitometry is shown in panel (c). (d, e) Empagliflozin inhibits OxLDL- or CT-1-induced MMP2 (d) and MMP9 (e) activity. SMCs made quiescent in medium supplemented with ITS-G 1x and no FBS were exposed to empagliflozin as in (a), followed by OxLDL (black) or CT-1 (red) for 18 hr and analyzed for MMP2 and MMP9 activity using SensoLyte® 520 fluorimetric assay. (f, g) Empagliflozin inhibits OxLDL- or CT-1-induced SMC proliferation and migration. Quiescent SMCs exposed to empagliflozin were treated with OxLDL (black) or CT-1 (red) for 48 hr (f) or 18 hr (g) and then analyzed for proliferation. (h) Empagliflozin reverses OxLDL-induced suppression of the mRNA expression of SMC markers *α*SMA and MYH11 and the proinflammatory markers ICAM1, Galectin 3, and Olr1 as analyzed by RT-qPCR. (a)  ^*∗*^*P* < 0.01 versus nLDL, ^†^*P* < 0.05 versus OxLDL (*n* = 5); (c)  ^*∗*^*P* < 0.05 versus nLDL, ^†^*P* < 0.05 versus OxLDL (*n* = 3); (d–g) *P* < 0.05 versus nLDL, ^†^*P* < 0.05 versus OxLDL or CT-1 (*n* = 4 or 5), (h)  ^*∗*^*P* < 0.05 versus nLDL, ^†^*P* < 0.05 versus OxLDL (*n* = 5).

Empagliflozin is an SGLT2 inhibitor and an FDA-approved antidiabetic drug. It has been shown to prevent glucose absorption by targeting SGLT2 expressed in the S1 segment of proximal tubules in kidneys [37–39]. Later, various clinical trials have demonstrated its cardioprotective effects independent of glucose absorption [40–43]. We and others have reported that aortic SMC expresses SGLT2 but at a lower level compared to kidney proximal tubule epithelial cells and whole kidneys [20, 21]. We also reported that empagliflozin inhibits IL-17A-induced SMC proliferation and migration by targeting TRAF3IP2/Reactive Oxygen Speciees (ROS)/NLRP3/Caspase-1-dependent IL-1*β* and IL-18 secretion [20]. Here, we investigated whether empagliflozin inhibits OxLDL-induced miR-30b expression, MMP activation, and SMC proliferation and migration. Moreover, we also determined whether empagliflozin blunts CT-1-induced MMP activation and SMC proliferation and migration. Indeed, pretreatment with empagliflozin at 1 mM concentration for 15 min attenuated OxLDL-induced mir-30b expression (Figure 7(a)) and restored RECK levels (Figure 7(b)). While panel 7B shows a representative western blot, the densitometric data from three to four experiments are shown in Figure 7(c). Further, empagliflozin at a similar concentration inhibited OxLD-induced MMP2 (Figure 7(d)) and MMP9 (Figure 7(e)) activation. Similarly, empagliflozin pretreatment attenuated CT-1-induced MMP2 (Figure 7(d)) and MMP9 (Figure 7(e)) activation. Importantly, empagliflozin inhibited OxLDL- and CT-1-induced SMC proliferation (Figure 7(f)) and migration (Figure 7(g), the inset shows representative images of Matrigel™ transwell invasion). Further, empagliflozin pretreatment restored OxLDL-induced suppression in SMA markers aSMA and MYH11 and inhibited the proinflammatory phenotype changes, as evidenced by the markedly reduced expression of ICAM1, Galectin 3, and Olr1 (Figure 7(h)). Together, these results indicate that empagliflozin inhibits OxLDL-induced miR-30b expression, MMP activation, and SMC proliferation, migration, and proinflammatory phenotype changes (Figure 7).

## 4. Discussion

**Figure 8:**
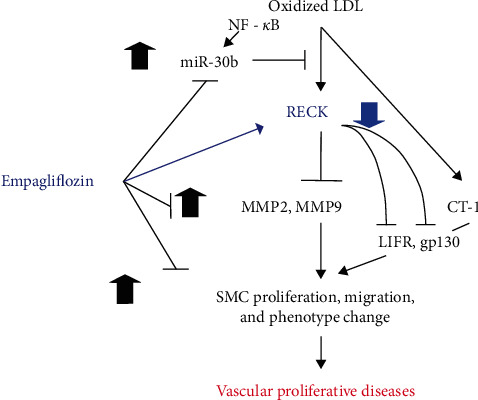
Schematic showing that pretreatment with empagliflozin inhibits OxLDL and CT-1-induced SMC proliferation, migration, and proinflammary phenotype changes. Exposure to OxLDL-induced NF-*κ*B-dependent miR-30b expression and miR-30b-mediated RECK suppression. Pretreatment with empagliflozin reversed these effects. Further, OxLDL induced MMP2 and MMP9 activation, and forced expression of RECK or pretreatment with empagliflozin blunted this response. OxLDL induced CT-1 expression and CT-1-stimulated SMC proliferation and migration in part via LIFR and gp130. Ectopic expression of RECK inhibited these effects by potentially associating with LIFR and gp130. Importantly, empagliflozin blunted CT-1-induced mitogenic and migratory effects. These results suggest the therapeutic potential of RECK overexpression or empagliflozin in vascular proliferative diseases.

Chronic inflammation and oxidative stress are features of vascular proliferative diseases. Our current study indicates that OxLDL, a major pathogenic factor in vascular proliferative diseases, downregulates RECK, an MMP regulator, in SMCs via oxidative stress-dependent and proinflammatory signaling pathways, leading to SMC migration, proliferation, and phenotype change. The causal role of RECK downregulation in promoting these mitogenic, migratory, and proinflammatory phenotype changes was further supported by studies showing that ectopic RECK overexpression effectively blunted these effects (Figure 8).

For the first time, we showed that OxLDL downregulates RECK expression in vascular SMCs, an effect that was mediated through the downregulation of its mRNA expression (Figure 1) via induction of miR-30b, an oxidative stress-responsive microRNA [36]. A search for potential targets of miR-30b (TargetScanHuman; https://www.targetscan.org/vert_80/; [44] identified the *Reck* gene as a potential target via a conserved 8mer site. In fact, our results show that transduction of a miR-30b-mimic downregulated RECK expression, whilst a miR-30b antagomir attenuated OxLDL-induced downregulation of RECK, strongly suggesting that miR-30b is a negative regulator of RECK.

Oxidative stress has been shown to upregulate miR-30b expression via an NF-*κ*B-dependent pathway [45, 46]. NF-*κ*B, a master regulator of inflammatory signaling pathways, is, in fact, regulated by oxidative stress [47]. It has been reported that NF-*κ*B, a redox-sensitive nuclear transcription factor, transcriptionally upregulates miR-30b [48] expression. Consistently, our results demonstrated that shRNA-mediated silencing of the p65 subunit of NF-*κ*B attenuated OxLDL-induced miR-30b upregulation (Figure 6(b)). All in all, our results suggest that OxLDL downregulates RECK via a redox-sensitive mechanism involving NF-*κ*B-dependent miR-30b induction.

Our study also revealed a novel mechanism by which empagliflozin inhibited SMC migration, proliferation, and proinflammatory phenotype changes. Various clinical trials have demonstrated the cardioprotective effects of empagliflozin, which are independent of its glucose-lowering properties. One of the key trials was the multicenter Empagliflozin, Cardiovascular Outcomes, and Mortality in Type 2 Diabetes trial (EMPA-REG OUTCOME) [41–43], which demonstrated a significant reduction in the rate of primary composite cardiovascular outcome and of death from any cause and slowed the progression of kidney disease in patients with type 2 diabetes and high cardiovascular risk [41, 43]. Other clinical trials have also supported the cardioprotective effects of empagliflozin, including the EMPEROR-Reduced [40] and EMPEROR-Preserved trials [49]. The EMPEROR-Reduced trial evaluated the effects of empagliflozin (10 mg once daily) or placebo in addition to recommended therapy in adults (≥18 years of age) who had chronic heart failure (functional class II, III, or IV) with a left ventricular ejection fraction of 40% or less. The trial outcome indicated that subjects who received empagliflozin had a lower risk of cardiovascular death or hospitalization for heart failure than those in the placebo group, regardless of the presence or absence of diabetes. EMPEROR-Preserved trials evaluated the potential beneficial effects of empagliflozin in patients with heart failure and a preserved ejection fraction, and trial outcome indicated that empagliflozin reduced the combined risk of cardiovascular death or hospitalization for heart failure, regardless of the presence or absence of diabetes. Overall, these clinical trials suggest that empagliflozin can be a beneficial treatment option for patients with type 2 diabetes and established cardiovascular diseases or heart failure.

Here, we showed that empagliflozin can effectively reverse OxLDL-induced downregulation of RECK, mediated by miR-30b inhibition (Figure 7). In fact, we have previously shown that empagliflozin inhibited IL-17A-induced oxidative stress via inhibition of NOX2- and NOX4-dependent ROS production [20]. Nicotinamide adenine dinucleotide phosphate hydrogen (NADPH) oxidases are the major source of ROS production induced by oxidized lipids [50–52]. Therefore, present data further support the antioxidant activity of empagliflozin against NADPH-oxidase-dependent ROS production. Indeed, clinical trial outcomes suggested empagliflozin's antioxidant potential as one of the underlying mechanisms for cardioprotective effects (Empagliflozin Multicenter International Randomized Parallel Group Double Blind Cardiovascular Safety Study [53]. Our results are in line with the trials' outcome and provide further mechanistic insights into its antioxidant and cardiovascular protective effects.

Empagliflozin is an inhibitor of SGLT2 in the kidney, which reduces glucose reabsorption and increases urinary glucose excretion, leading to improved glycemic control in patients with type 2 diabetes mellitus. While empagliflozin's primary mechanism of action is through the inhibition of SGLT2 in the kidney, it has also been shown to affect intracellular signaling pathways in various cell types. For instance, empagliflozin has been reported to activate adenosine monophosphate-activated protein kinase (AMPK) in the liver ameliorating lipid accumulation in obesity-related nonalcoholic fatty liver disease [54] and in skeletal muscle promoting fat utilization and hence presumably contributing to improved insulin sensitivity [55, 56]. Additionally, empagliflozin-activation of AMPK in cardiomyocytes has been shown to reduce lipopolysaccharide (LPS)-induced inflammatory response and counteract LPS-impaired cellular ATP/ADP ratio, suggesting potential mechanisms for cardioprotection. Empagliflozin has also been suggested to directly (i.e., independent of SGLT2) inhibit cardiac Na+/H+ exchanger (NHE) flux and thus causing reduction of cytosolic Na+ and Ca^2+^ concentrations while elevating mitochondrial Ca^2+^ concentration, which could be a part of underlying mechanisms of empagliflozin's cardioprotective effects [57]. NHE inhibition is also evident in vascular endothelial cells, leading to a reduction of oxidative stress that confers vasculoprotective effects [58]. Therefore, empagliflozin's effects on these signaling pathways suggest that it may have beneficial effects beyond its glucose-lowering effects in patients with type 2 diabetes mellitus.

Empagliflozin's antioxidant activity is consistent with the downregulation of miR-30b through inhibition of NADPH-oxidase-dependent ROS production; however, it is unclear how empagliflozin could regulate NADPH-oxidase activity. In our previous study [20], we demonstrated that empagliflozin blocks the NLRP3-dependent proinflammatory signaling pathway in SMCs, which is mediated through SGLT2-independent mechanisms, as silencing SGLT2 did not abrogate its effects. In fact, other studies [59] also reported that SGLT2 inhibitors, including Empagliflozin, may express SGLT2-independent effects but via sodium/hydrogen exchanger 1 (NHE-1), as discussed above. Of note, vascular SMCs express NHE-1, and inhibition of NHE-1 by amiloride or dimethyl amiloride blunts lysophosphatidic acid-induced vascular SMC proliferation [60]. Therefore, it appears that Empagliflozin and other SGLT2 inhibitors act via SGLT2 and/or NHE in SMCs. Intriguingly, it has been reported that pharmacological inhibition or genetic haploinsufficiency of NHE-1 lowers NADPH-oxidase activation in ischemic brains [61], which is likely caused by intracellular pH reduction due to decreased Na/H exchange [62]. Therefore, our current results combined with our previous findings, suggest that empagliflozin has a direct effect on SMCs via SGLT2-independent and antioxidant mechanisms, apart from its glycemic control effects.

In addition to antioxidant effects, our results also show that empagliflozin exerts anti-inflammatory effects mediated through RECK induction and downregulation of CT-1-dependent signaling. CT-1 is a member of the IL-6 family of cytokines and was originally identified as a factor that induces a hypertrophic response in neonatal cardiac myocytes. It also promotes cardiomyocyte maturation and protects against cardiomyocyte death during ischemic injury, demonstrating its pleiotropic effects [63, 64]. On the other hand, it has been shown to play a proatherogenic role in arterial tissue [65]. In line with our observations, that study [65] indicated that CT-1 promotes migration, proliferation, and the production of type I collagen in human aortic SMCs. More importantly, continuous infusion of CT-1 promoted atherosclerosis in Apoe-deficient mouse, a widely used animal model of atherosclerosis, and the effect was attributed to increased macrophage invasion and elevated SMC proliferation and collagen deposition [65], further supporting the causative role of increased CT-1 in promoting vascular proliferative diseases.

CT-1 signals through its cognate receptor, CT-1-specific alpha chain receptor, which interacts with LIFR upon ligand binding and recruitment of gp130. It has also been shown that CT-1 directly binds to LIFR, and subsequent recruitment of gp130 activates intracellular signaling cascades, mediated by STAT3-dependent and also MAPK-dependent pathways. Consistent with the notion, shRNAs or neutralizing antibodies against LIFR and gp130 attenuated CT-1-induced SMC migration and proliferation. Importantly, forced expression of RECK blunted CT-1-induced proliferation and migration (Figures 5(a) and 5(b)), indicating that RECK can negatively regulate CT-1-signaling activity. In fact, our result indicates that RECK can be co-immunoprecipitated with LIFR and gp130 (Figure 5), suggesting a physical interaction of the three molecules. It is tempting to speculate that RECK negatively regulates LIFR and gp130-initiating signaling via direct interaction with the complex, suggesting a novel function of RECK as a regulator of proinflammatory signaling.

Our results also show that empagliflozin lowered MMP2 and MMP9 activities, consistent with RECK upregulation, leading ultimately to inhibition of SMC migration and proliferation. MMPs play a critical role in matrix protein metabolism, tissue homeostasis, and pathogenic tissue remodeling [4, 66]. Supporting earlier reports [67, 68], here we show that OxLDL elevated MMP2 and MMP9 activities in SMCs, which coincided with enhanced migration and proliferation (Figure 2). Our results also indicate that the downregulation of RECK by OxLDL might have contributed to elevated MMP2 and MMP9 activities, as RECK is a potent MMP2 and MMP9 inhibitor [10, 12]. Moreover, empagliflozin suppressed MMP2 and MMP9 activities in OxLDL- or CT-1-exposed SMCs, consistent with empagliflozin-induced RECK upregulation and, thus RECK-mediated MMP2 and MMP9 inhibition. Importantly, our data also show that Empagliflozin reverses OxLDL-induced SMC phenotype changes, as evidenced by the reversal of OxLDL-induced suppression of SMC markers *α*SMA and MYH11, and the proinflammatory markers ICAM1, Galectin 3 and Olr1. These results suggest that RECK induction of Empagliflozin treatment has broader protective effects that need further investigation.

In summary, our current study identified a novel mechanism by which oxLDL promotes SMC migration and proliferation., i.e., via NF-*κ*B-dependent upregulation of miR-30b and inhibition of RECK. Another significant finding is that exposure to Empagliflozin counteracts oxLDL-induced SMC migration and proliferation via RECK induction. Moreover, OxLDL induced CT-1 expression and CT-1-stimulated SMC proliferation and migration in part via LIFR and gp130. Ectopic expression of RECK inhibited these effects by physically associating with LIFR and gp130. Importantly, empagliflozin blunted CT-1-induced mitogenic and migratory effects. These results suggest the therapeutic potential of RECK overexpression or Empagliflozin in vascular proliferative diseases (Figure 8).

## Data Availability

The data used to support the findings of this study are included within the article.

## Abbreviations

ACTA: Actin alpha 2, smooth muscle
MYH11: Myosin heavy chain 11
OxLDL: Oxidized low-density lipoproteins
nLDL: Native low-density lipoproteins
HASMC: Human aortic smooth muscle cells
RECK: Reversion-inducing cysteine-rich protein with Kazal motifs
miR: Micro RNA
CT-1: Cardiotrophin-1
LIFR: Leukemia inhibitory factor receptor alpha
aLIFR-Ab: Anti-LIFR antibody
gp130: Membrane glycoprotein 130
IL6ST: Interleukin 6 cytokine family signal transducer
siRNA: Small interfering RNA
shRNA: Short hairpin RNA
GFP: Green fluorescent protein
eGFP: Enhanced GFP
NC: Negative control for miRNA
IP: Immunoprecipitation
IB: Immunoblotting
MMP: Matrix metallopeptidase
DMSO: Dimethylsulfoxide
GPI: Glycosylphosphatidylinositol-anchored proteins
ITS: Insulin–transferrin–selenium
ICAM1: Intercellular adhesion molecule 1
LGALS3: Lectin, galactoside-binding, soluble
Olr1: Oxidized low-density lipoprotein receptor 1.
